# Effect of hyperthermia on intestinal microecology, immune function, and progression-free survival in patients with advanced unresectable lung adenocarcinoma

**DOI:** 10.1038/s41598-023-44350-w

**Published:** 2023-10-11

**Authors:** Jin Tian, Lin Long, Jianhua Zang, Wei Liu, Peng Liu, Lili Zhao, Xin Liang, Jun Xiao

**Affiliations:** 1 Oncology Center I Department, Qingdao Hiser Hospital Affiliated of Qingdao University (Qingdao Traditional Chinese Medicine Hospital), Shibei District, No. 4 Renmin Road, Qingdao, 266033 Shandong China; 2 Department of Radiotherapy, Oncology Center, Qingdao Hiser Hospital Affiliated of Qingdao University (Qingdao Traditional Chinese Medicine Hospital), Qingdao, 266033 Shandong China; 3 Outpatient Department (or Office), Qingdao Hiser Hospital Affiliated of Qingdao University (Qingdao Traditional Chinese Medicine Hospital), Shibei District, No. 4 Renmin Road, Qingdao, 266033 Shandong China

**Keywords:** Cancer, Lung cancer

## Abstract

This study aims to investigate the effects of hyperthermia on intestinal microecology, immune function, and progression-free survival of patients with advanced unresectable lung adenocarcinoma. A total of twenty patients with lung adenocarcinoma in the study group received the advanced standard first-line treatment protocol, which included pemetrexed + cisplatin combined with sintilimab immunotherapy and hyperthermia. Additionally, twenty patients with lung adenocarcinoma in the control group received the advanced standard first-line treatment protocol, which included pemetrexed + cisplatin combined with sintilimab immunotherapy. The T-lymphocyte subpopulation and CD4/CD8 cell ratio of each sample were detected using flow cytometry. The intestinal flora was evaluated using 16S rRNA gene sequencing. The study observed the changes in the abundance, distribution, composition, and structure of fecal gut microorganisms before and after the treatment in both groups of patients. Significant differences were observed in the intestinal flora between the two groups. The patients in the study group showed improved immunity after treatment, whereas there was no significant change in the immunity of the control group before and after treatment. However, the difference in progression-free survival between the two groups was not statistically significant. Hyperthermia has a significant impact on improving the microecology of intestinal flora and the immunity of patients, but it does not have a significant effect on prolonging the progression-free survival of patients.

## Introduction

The most frequent type of cancer and the main cause for cancer-related mortality globally is lung cancer. As of the latest available statistics up to 2023, approximately 2 million new cases of lung cancer are diagnosed worldwide each year^1^. A significant portion of lung cancers are still diagnosed with mid- to late-stage tumors, and the 5-year survival rate is only 18%^2^, resulting in a significant disease burden for the world. Lung cancer incidence has been rising annually^3^, and despite the advances made by researchers and clinicians in tumor diagnosis and treatment in recent decades.

Hyperthermia, a therapeutic technique that raises the temperature of specific body areas or the entire body beyond the normal 37 °C (98.6°F) for medical purposes, holds a diverse range of applications. In cancer therapy, it plays a pivotal role by damaging cancer cells directly, sensitizing them to radiation or chemotherapy, boosting local blood flow, enhancing oxygen and nutrient delivery, stimulating the immune system, and instigating programmed cell death in select cancer cells. Hyperthermia's temperature ranges, from mild (around 40–42 °C) to moderate (42–45 °C), are carefully chosen based on clinical context and treatment objectives, with extreme hyperthermia (above 45 °C) avoided due to tissue damage risks. Administered by healthcare professionals using specialized equipment, hyperthermia is a significant adjuvant therapy, considering the differing responses of tumor and normal tissues to elevated temperatures. Its effects encompass direct tumor cell destruction, modulation of the tumor microenvironment, induction of heat-shock proteins, DNA damage, interference with DNA repair pathways, initiation of apoptotic cascades, and promotion of tumor neoantigen production, making it a versatile tool in cancer treatment^4^.

The gut microbiota has been demonstrated to have an impact on various metabolic traits, including energy regulation from diet, systemic inflammation, gut barrier function, and insulin sensitivity^5,6^. These metabolic traits are known to be altered during the development of cancer. Moreover, recent animal studies have indicated that the gut microbiota can affect tumor development and the effectiveness of cancer therapies^7–9^. Environmental factors play a crucial role in maintaining the balance of the microbiota, and alterations in body temperature significantly impact its functioning. The sensitivity of the intestinal epithelium to temperature makes changes in body temperature particularly influential. In their study, Juang et al.^10^ determined that the average heat dose applied to tumors was 21.3 ± 16.5 CEM43℃, whereas the rectal heat dose was 1.6 ± 1.2 CEM43℃ (CEM43℃ represents cumulative equivalent minutes at 43℃, a standardized unit for measuring heat exposure and damage). These findings indicate that although the majority of the heat dose is targeted towards the tumor, there are also temperature changes occurring in the surrounding area, including the gastrointestinal tract. In addition to direct local heating, the temperature of the surrounding tissues is also increased due to the heating of normal blood flowing through the tumor. Consequently, the heat is transferred to the colder tissues nearby. Therefore, it is probable that the heat therapy has an impact on the microbiota.

In 2007, the Human Microbiome Project, initiated by the National Institutes of Health of the U.S.A, utilized 16S rRNA sequencing technology as one of its main technical methods. Since then, this technology has been widely employed in the classification and identification of intestinal flora. The key features of 16S rRNA sequencing technology are its accuracy, rapidity, and sensitivity, which allow for a comprehensive understanding of the species, distribution, and abundance of the intestinal flora. This technology effectively addresses the limitations of traditional methods such as morphology examination, isolation, and culture, which are often time-consuming and complex. Consequently, it has become a standard technique for analyzing the intestinal microbiota in humans and is now widely used.

The effects of different forms of hyperthermia, such as intraperitoneal thermal chemotherapy (HIPEC), deep regional and systemic hyperthermia, on the microbiota have not been fully understood. Both preclinical and clinical evidence suggests that hyperthermia can inhibit tumor growth in the long term, either alone or in combination with other therapies. However, it’s important to note that the severity of hyperthermia and its side effects can vary depending on the underlying cause and the individual's overall health, such as burns or blisters. Besides, there have been fewer studies conducted to determine whether hyperthermia has a positive or negative impact on the intestinal flora. Considering the potential of temperature changes to influence the function and composition of gut microbes, as well as the impact of gut flora on tumorigenesis and development, we conducted this study to generate clinically significant implications for the treatment of lung cancer.

## Materials and methods

1. Study Subjects: A total of 40 patients with advanced lung adenocarcinoma were recruited from January 1, 2022, to January 1, 2023, at the Oncology Center of Qingdao Hiser Hospital, affiliated with Qingdao University. The study protocol underwent review and was approved by the Ethics Committee of Qingdao Hiser Hospital affiliated with Qingdao University. We confirm that all methods were performed in accordance with the relevant guidelines and regulations.
2. The diagnostic criteria for patients with inoperable stage IV driver-negative lung adenocarcinoma involve the use of definitive pathology, relevant immunohistochemistry, and genetic testing to confirm the diagnosis.
3. The inclusion criteria for this study were as follows: ① primary patients (EGFR wild type) who met the diagnostic criteria and were not receiving any treatment; ② individuals aged between 18 and 65 years, with no gender restrictions; ③ participants who agreed to participate and signed the informed consent form; ④ genetic test results indicating that the PD-L1 expression rate (Tumor Cell Positive Proportion Score TPS) was equal to or less than 49%.
4. The following exclusion criteria were applied in this study: ① individuals with serious respiratory, circulatory, digestive, urinary, endocrine, hematologic, neurological, or psychiatric diseases; ② pregnant and lactating females; ③ individuals with concurrent tumors; ④ individuals who have taken antibiotics or microecological agents within the past month; ⑤ patients who can undergo early-stage surgery or receive radical radiotherapy; ⑥ patients who test positive for the driver gene.
5. Discontinuation and dropout criteria include: (1) individuals using drugs that are prohibited by the program; (2) individuals experiencing serious adverse events; (3) individuals who have not completed a sufficient number of observation cycles; and (4) individuals who have missed a visit during the follow-up period.
6. Treatment Regimen: The study group received a combination of 500 mg/m^2^ pemetrexed + 75 mg/m^2^ cisplatin along with 200 mg sindilizumab and underwent tumor hyperthermia during the treatment period. On the other hand, the control group received 500 mg/m^2^ pemetrexed + 500 mg/m^2^ cisplatin along with 200 mg sindilizumab but did not receive any additional antitumor regimen.
7. Tumor hyperthermia implementation plan: (1) Preheat the HY-7000I radiofrequency hyperthermia machine (Nanjing Hengpu Weiye Science and Technology Co., Ltd.) for 3–5 min before starting the hyperthermia. (2) Choose the appropriate pole plate based on the target area for hyperthermia. (3) The patient should lie on the treatment bed either supine or prone, aligning the upper and lower pole plates with the treatment area. The recommended distance between the pole plates and the treatment area is 5–7 cm. (4) The patient should keep the treatment area still throughout the treatment process. (5) Within five minutes of turning on the heat treatment machine, adjust the temperature to 43.5 ℃ and maintain it for 45 min.

The treatment was implemented by targeting the site where the tumor exists. Hyperthermia was administered during or immediately after chemotherapy. The target area received hyperthermia twice a week during chemotherapy, with a time interval between the two sessions ranging from 48 to 72 h. Each course of treatment consisted of eight sessions of hyperthermia. The power used during treatment was adjusted based on the patient's feedback, with a minimum power of 500 watts and a maximum power of 600 watts. Each session lasted for 45 min. During the treatment period, the patient's heart rate and blood pressure were recorded every 15 min. Any side effects experienced by the patient, such as discomfort, pain, burns, or interruptions in the treatment, were also recorded.

### Observation indicators

#### Immune function testing

T cell subsets CD3+ CD4+ and CD3+ CD8+ were detected before treatment and at the end of 4 cycles of treatment. To avoid light staining, 50 μL of whole blood was taken and mixed with 20 μL of anti-PE-CD4 (Batch No.: 2123689, Invitrogen) and anti-APC-CD8 antibody (Batch No.: 2247551, Invitrogen) at room temperature for 20 min. The mixture was then added to 450 μL of 1 × FACS Calibur flow cytometer for detection. T lymphocyte counting was performed using MultiSET software.

#### Intestinal flora testing

About 3 g of fecal samples were collected from patients before treatment and 24 h after 4 cycles of treatment. The composition of their intestinal microorganisms was examined using 16S rRNA gene sequencing. The specific methods followed were as follows: Before collecting feces, subjects were instructed to empty their bladder to prevent sample contamination. The researcher wore disposable sterile gloves and collected fresh uncontaminated feces from the middle and inner parts of the subjects. The collected feces were then loaded into fecal genome protection solution (Ovison Genetic Technology Co., Ltd.) for pretreatment. The samples were divided into 3 equal portions and sealed in sterile freezing tubes. They were subsequently transferred to liquid nitrogen for storage until further analysis. The 16S rRNA sequencing work was conducted by Ovison Genetic Technology Co., and the resulting data were analyzed by the same company. In summary, 16S rRNA gene sequencing was performed by Ovison Gene Technology Co. in this study.

#### Progression-free survival time (PFS)

The time from the diagnosis of lung adenocarcinoma to the progression of the patient's disease, such as enlargement of the lesion or the appearance of distant metastases, was recorded. The scoring criteria according to RECIST 1.1 were used. Subsequently, a survival curve was plotted. The log-rank test was then performed to compare the progression-free survival between the two groups.

### Statistical methodology

All data were analyzed using SPSS 26.0. Descriptive statistics were reported as mean ± standard deviation (x̅ ± s). The paired-samples t-test was employed for variables that followed a normal distribution, whereas the rank-sum test was used for variables that did not conform to a normal distribution. A p-value of less than 0.05 was considered statistically significant. Progression-free survival curves were generated using the Kaplan–Meier method, and the log-rank test was utilized to compare differences in progression-free survival between the two groups.

### Ethical approval and consent to participate

Our study was approved by the Ethics Committee of Qingdao Hiser Hospital Affiliated of Qingdao University(Qingdao Traditional Chinese Medicine Hospital).

## Results

### Immune function

In the study group, the proportion of CD4+ T cell counts significantly increased after 4 cycles of treatment, whereas the level of CD8+ T cells decreased compared to before treatment in the study group(*P* < 0.05). However, the proportion of CD8+ T cells in the control group significantly decreased before and after treatment, which could be attributed to the application of anti-tumor immunotherapy. These findings indicate that hyperthermia can effectively regulate the distribution of peripheral blood T-cell subpopulations. For more details, refer to Tables 1 and 2.

**Table 1:** Comparison of T-lymphocyte subpopulation expression rates before and after treatment in 20 patients in the study group (x̅ ± s).

Detection indicators	Study group before treatment	Study group after treatment	T	P
CD4^+^T (cells/mL)	560.20 ± 326.17	744.15 ± 305.14	− 2.47	0.023
CD8^+^T (cells/mL)	747.65 ± 304.05	519.55 ± 230.07	4.80	< 0.001
CD4/CD8	1.41 ± 0.88	1.61 ± 0.76	− 1.15	0.27

**Table 2:** Comparison of cell expression rates of T lymphocyte subpopulations before and after treatment in 20 patients in the control group (x̅ ± s).

Detection indicators	Control group before treatment	Control group after treatment	T	P
CD4^+^T (cells/mL)	589.15 ± 338.58	640.25 ± 335.06	− 1.93	0.068
CD8^+^T (cells/mL)	685.90 ± 320.16	553.95 ± 267.69	2.57	0.019
CD4/CD8	1.40 ± 0.81	1.37 ± 0.83	0.25	0.809

### Intestinal flora

The specimens in the control group before treatment were named HLA1, and the specimens after treatment were named HLA2. Similarly, the specimens in the study group before treatment were named HLB1, and the specimens after treatment were named HLB2.

#### General information

**Figure 1:**
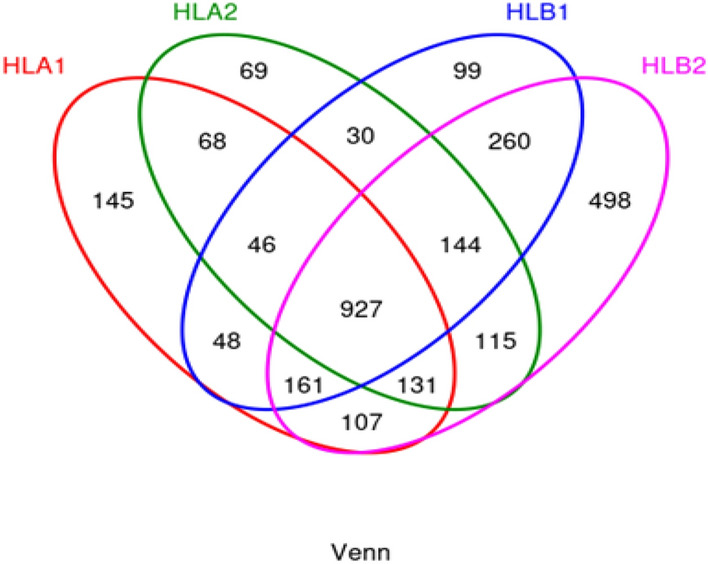
Venn diagram illustrating the distribution of Operational Taxonomic Units (OTUs). Each color represents a different sample. The overlapping area of the circles indicates the intersection of OTUs present in multiple samples, whereas the non-overlapping regions represent unique OTUs.

The cluster analysis of Operational Taxonomic Units (OTUs) resulted in a total of 2989 OTUs. After the draw leveling process, 2848 OTUs remained. Among these, the HLA1 group had 145 OTUs, the HLA2 group had 69 OTUs, the HLB1 group had 99 OTUs, and the HLB2 group had 498 OTUs. In total, there were 2037 common OTUs. Please refer to Fig. 1 for a visual representation.

#### Alpha diversity

**Figure 2:**
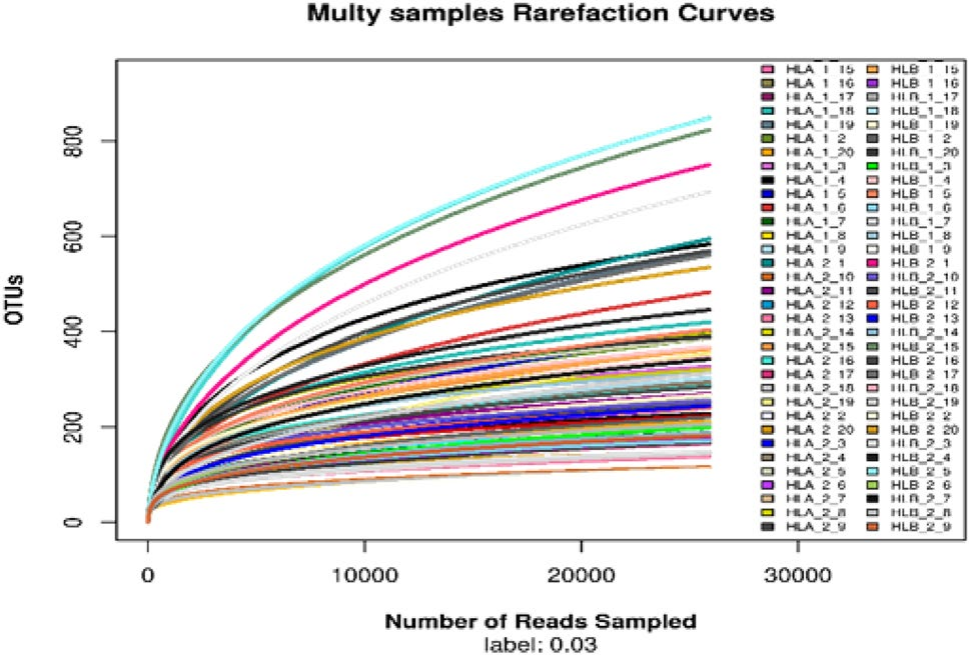
Rarefaction curve.

The sparse curve (Fig. 2) illustrates a gradual increase in the total number of observed species as the number of sequenced sample sequences increases. This indicates that the current sequencing results adequately capture the diversity of the samples. Thus, the amount of sequencing data is considered reasonable, reflecting a high degree of sample diversity.

##### Shannon–Wiener curve

**Figure 3:**
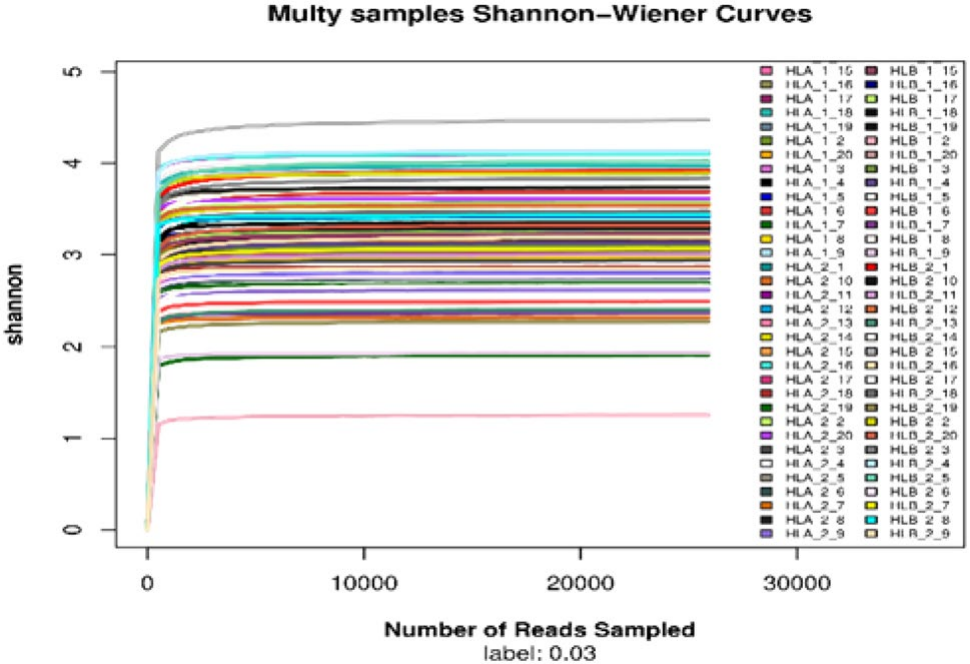
The Shannon curve.

The Shannon curve (Fig. 3) gradually flattens out, indicating that the sequencing data is sufficient to capture the information of the majority of microorganisms present in the sample.

##### The alpha diversity index

Chao1 is a strain richness index that is used to estimate the number of OTUs in the community. Simpson's index combines richness and evenness, and is used to estimate one of the indices of microbial diversity in the samples. PD_whole_tree is a genealogical diversity index that takes into account the abundance of the species as well as the distance of evolution. A higher value indicates higher community diversity. Shannon index is another index used to estimate microbial diversity in the samples, and a higher Shannon value indicates higher community diversity. Observed_species refers to the number of OTUs actually observed with the increase of sequencing depth.

**Figure 4:**
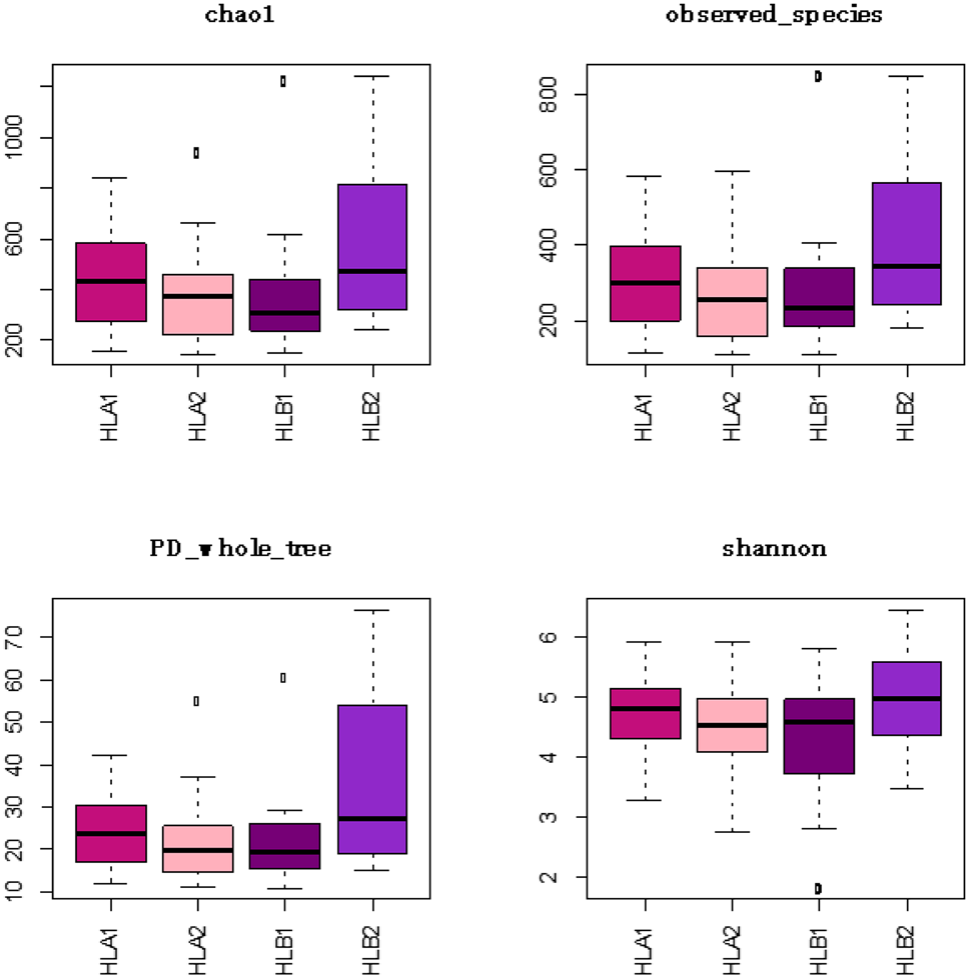
Box plot of alpha diversity index.

Alpha diversity, which represents the variation in taxonomic diversity within the samples, was measured first. Our analysis revealed statistically significant differences in three diversity indices (chao1, observed_species, and PD_whole_tree) between the two post-treatment samples and the pre- and post-treatment samples of the study group (Fig. 4 and Table 3). These findings suggest that the diversity of human intestinal flora in patients from the study group significantly differed after treatment compared to patients in the control group after treatment. Furthermore, the intestinal flora of patients in the study group exhibited changes before and after treatment.

**Table 3:** Comparison of community richness and diversity among groups.

Group	A1–A2	B1–B2	A1–B1	A1–B2	A2–B1	A2–B2
Chao1 (diff)	−73.56	218.22	−67.49	150.73	6.07	224.29
*P*	0.79	0.04	0.83	0.24	0.99	0.03
Simpson (diff)	−0.02	0.04	−0.05	−0.01	−0.03	0.01
*P*	0.91	0.51	0.36	0.99	0.76	0.98
PD_whole_tree (diff)	−2.23	13.50	−2.53	10.97	−0.30	13.21
*P*	0.95	0.01	0.93	0.05	0.99	0.01
Shannon (diff)	−0.23	0.53	−0.33	0.20	−0.10	0.43
*P*	0.80	0.18	0.58	0.88	0.98	0.35
observed_species (diff)	−50.67	142.46	−37.99	104.47	12.68	155.14
*P*	0.76	0.04	0.88	0.19	0.99	0.02
goods_coverage (diff)	0.001	−0.003	0.002	−0.002	0.001	−0.003
*P*	0.87	0.11	0.66	0.66	0.98	0.23

#### Beta diversity

**Figure 5:**
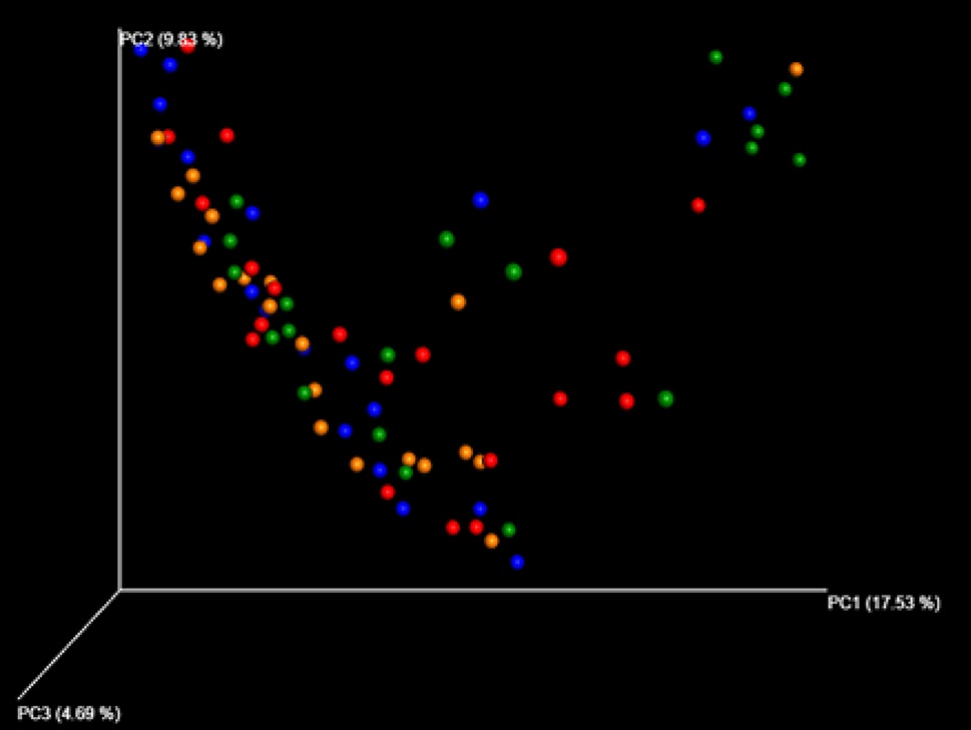
Unweighted PCoA based 3D map.

Microbial community intergroup variability (Beta diversity) was assessed using unweighted UniFrac distances. Principal Coordinate Analysis (PCoA) was performed based on these distances, and potential principal components influencing differences in the composition of the sample communities were identified through dimensionality reduction. The PCoA plots in Fig. 5 demonstrate the separation of the two groups. A significant difference (p = 0.035) was observed in the colony structures of the two groups. The results further indicated that the structural diversity of the gut microbiota was significantly influenced by hyperthermia, as it differed between the treatment and control patients.

**Figure 6:**
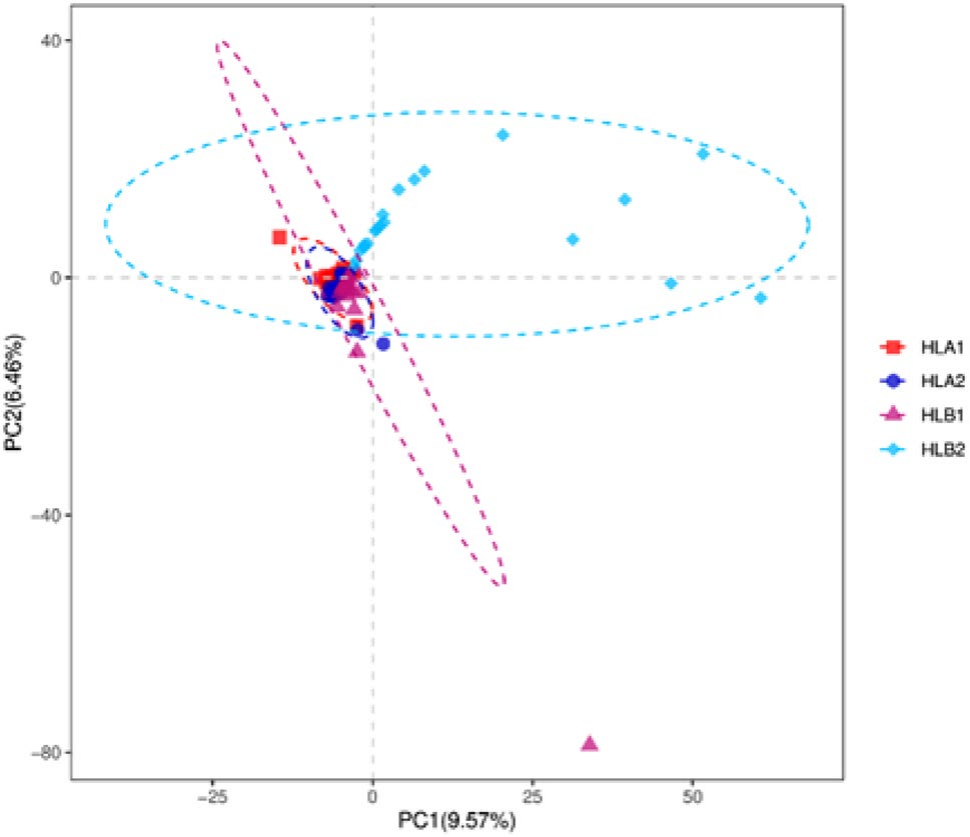
PLS-DA analysis revealed that the study group showed no overlap with the samples from the other group after undergoing hyperthermia treatment. This suggests a significant alteration in the microbial community of the samples following the treatment.

Partial Least Squares Discrimination Analysis (PLS-DA) (Fig. 6) is a multivariate statistical method used for discriminant analysis. Discriminant analysis is a common statistical technique used to categorize research objects based on observed or measured variables. PLS-DA is a supervised method that models the relationship between microbial content and sample categories to predict the category of a given sample.

#### LEFse analysis

To assess the contribution of differential strains to the observed differences, we employed the LEfSe method for analyzing these strains. We compared the changes in bacterial flora at six taxonomic levels (Phylum, Class, Order, Family, Genus, and Species) before and after treatment in both patient groups. Our analysis focused on identifying the important strains that exhibited substantial differences before and after treatment. The results were presented in a histogram, depicting the distribution of LDA values, with a threshold set at 3. Different colors represented different groups, and the length of each bar indicated the magnitude of the contribution of the respective strains to the observed differences. Longer bars indicated greater contribution. Taxonomic units exceeding the default threshold were considered to have significant differences in abundance between groups. The magnitude of the value corresponded to the significance level, with higher values indicating greater significance.

**Figure 7:**
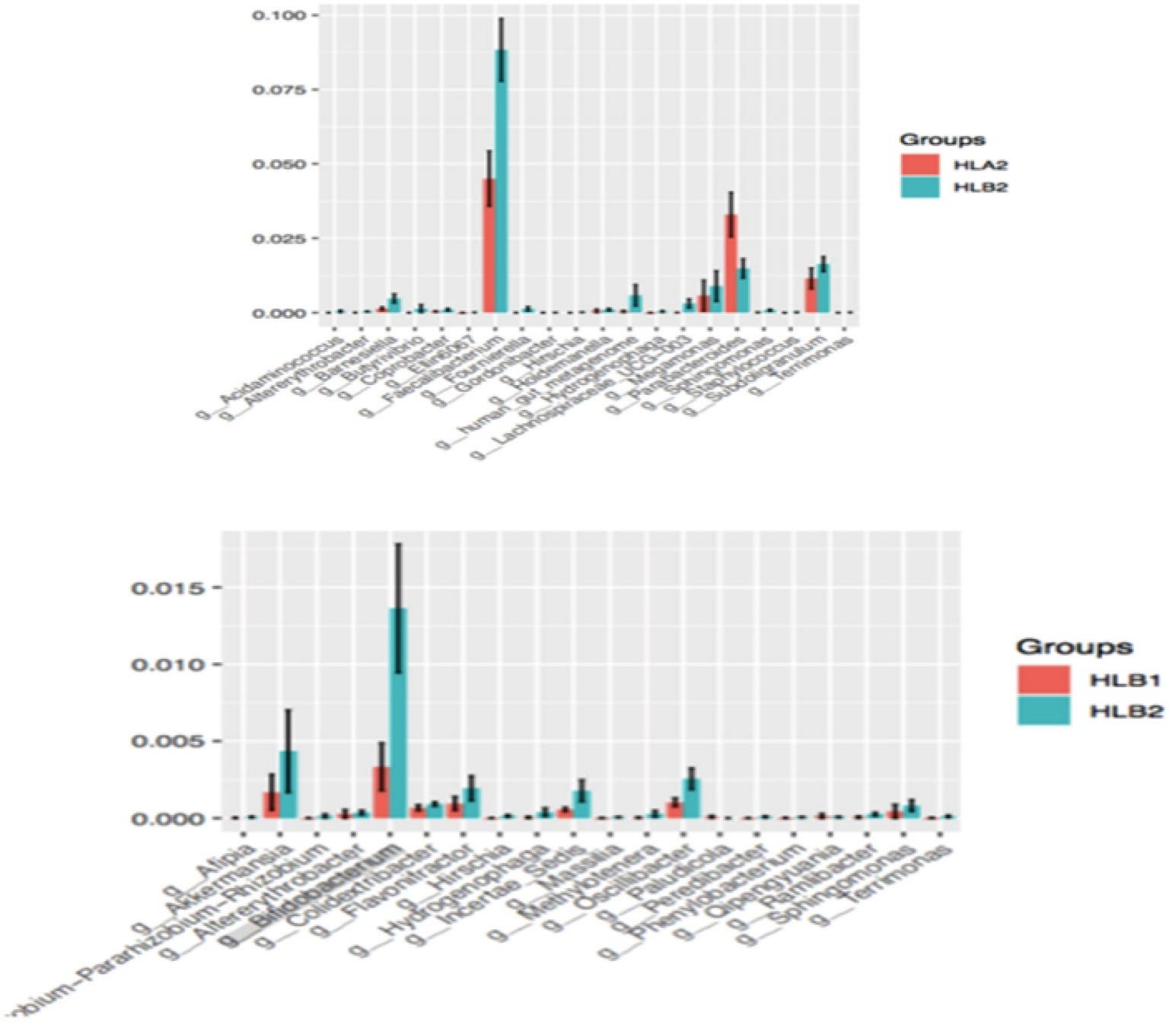
Histogram of species differences in different groups.

**Figure 8:**
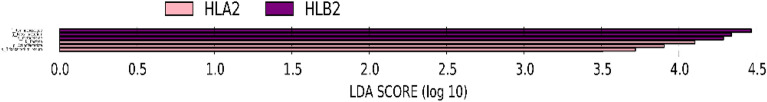
Histogram of LDA distribution for LEfSe analysis based on categorical information.

The results indicated a significant difference in bacterial species between the two groups before and after treatment. HLA1 showed a significantly higher abundance compared to HLA2, specifically in the bacterial taxa p__Cyanobacteria and g__Faecalibacterium. On the other hand, HLB2 exhibited a significantly higher abundance than HLB1 in the bacterial taxon g__Bifidobacterium. Additionally, HLB2 showed a significantly higher abundance than HLA2 in the bacterial taxa g__Faecalibacterium and g__Subdoligranulum, whereas it had a significantly lower abundance in the bacterial taxon g__Parabacteroides. HLB2 was characterized by the presence of the family Ruminococcaceae and the genus Faecalibacterium. Furthermore, HLB2 had a significantly lower abundance than HLA2 in the bacterial taxon g__Parabacteroides. HLB2 exhibited a higher abundance in the family Ruminococcaceae and the genus Faecalibacterium. On the other hand, HLA2 showed a higher abundance in the phylum Actinobacteriota and the species Bifidobacterium longum (see Figs. 7, 8).

### Survival analysis

**Figure 9:**
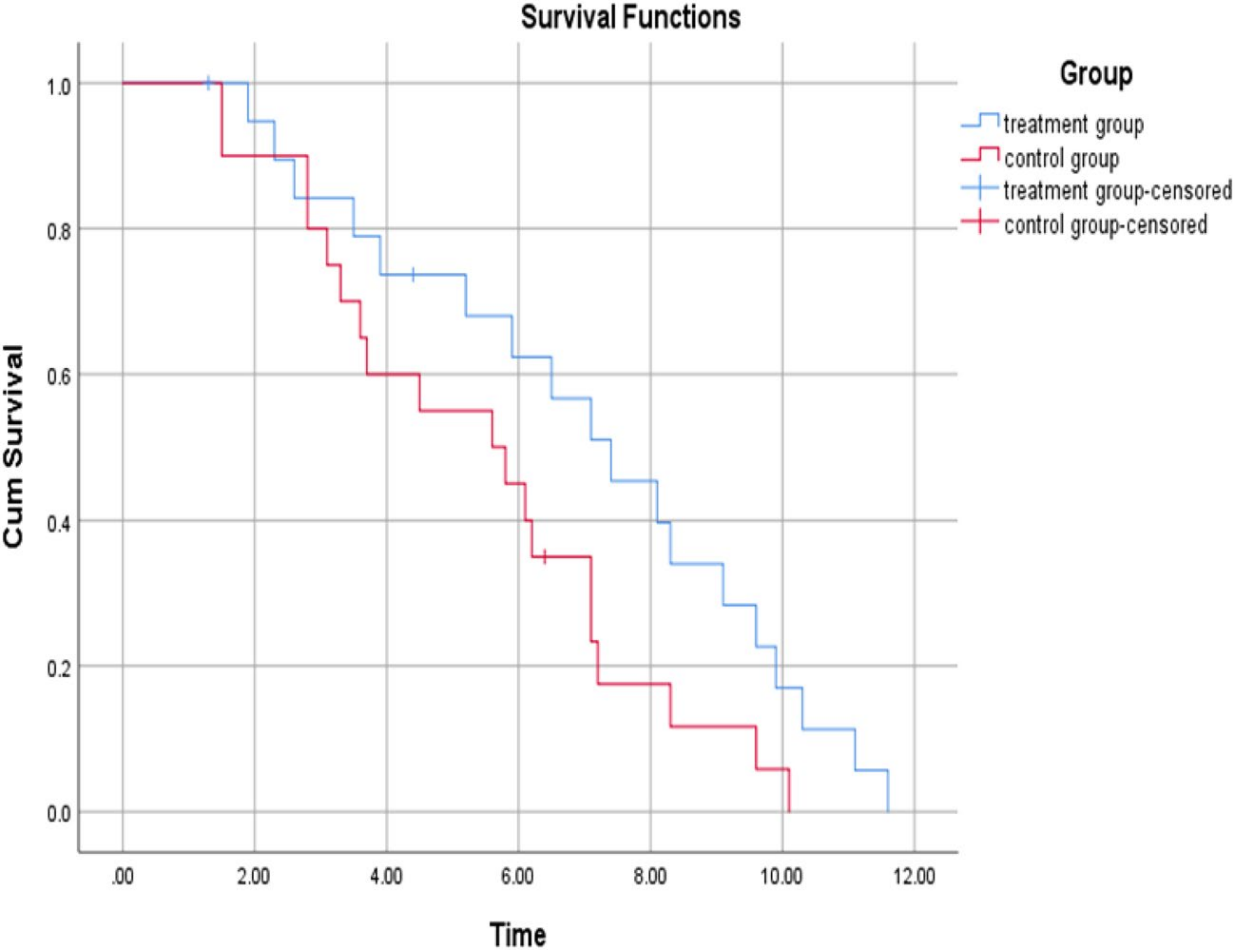
PFS survival curves for both groups of patients.

In the study group, there were 2 cases of censored data, including 2 patients who were lost to follow-up. No patients withdrew from the study or stopped observation because of the study time limit. The mean PFS in the study group was 6.99 months, with a median of 7.4 months. In the control group, there was 1 case of censored data, which involved 1 lost patient. No patients withdrew from the study or stopped observation due to the study timeframe. The mean PFS in the control group was 5.41 months, and the median PFS was 5.6 months. Statistical analysis indicated that there was no statistically significant difference in PFS between the two groups (*P* > 0.05) (see Fig. 9, Table4).

**Table 4:** Statistical difference in PFS between the two groups of patients.

Group	Average value	Median	χ^2^	*P*
PFS in study group (months)	6.99	7.4	3.57	0.059
PFS in control group (months)	5.41	5.6		

## Discussion

The intestinal tract of healthy individuals contains a vast number of microbial species, including approximately 1800 genera and 40,000 strains of bacteria. These bacteria possess 100 times more genes than humans^11^. These intricate microbial communities have established a harmonious symbiotic relationship with the host throughout its life cycle^12^. They are closely associated with the development of the host's immune system, nutrient absorption and distribution, drug metabolism, and overall health^13^. Due to their significant impact, they are often referred to as the "forgotten organ"^14^. The co-evolution and regulatory interactions between the microbiota and the immune system are well-established^15^. However, our understanding of how this relationship influences tumor development and therapeutic response is still in its early stages.

The mechanism of hyperthermia in treating tumors involves not only the direct killing of tumor cells through heat, but also the synergistic effects it has with radiotherapy and chemotherapy. Additionally, hyperthermia plays a crucial role in enhancing the body's immune response and inducing a specific anti-tumor effect. This is because the antigen related to tumor immunity is mainly present on the cell surface, and heat therapy can modify the fluidity of the tumor cell membrane's lipid bilayer, thereby activating the anti-tumor cellular immune response. Moreover, hyperthermia can disrupt the inhibitory effects of tumor cell-secreted sealing factor and macrophage movement inhibitory factor on the immune system, allowing the body to regain its immune response against the tumor^16,17^. Previous studies^18^ have indicated that patients with tumors often experience varying degrees of immune deficiencies, characterized by a decrease in CD4+ T cells, an increase in CD8+ T cells, and a decrease in the CD4+/CD8+ ratio. These immune deficiencies allow tumors to evade immune surveillance and promote tumor growth. In a study conducted by Stawarz et al.^19^, it was found that local hyperthermia can lead to a certain level of improvement in the patient's CD4+/CD8+ ratio compared to the ratio before treatment. This improvement suggests an enhancement in the body's immune function to some extent. Additionally, there is evidence demonstrating that exposing lymphokine-activated killer cells to temperatures above 45 degrees Celsius significantly enhances their tumor-killing activity^20^. Furthermore, this temperature exposure also enhances the immune effects of macrophages, lymphocytes, and other cells^21^.

In this study, the study group showed higher levels of CD4+ T cells and CD4+/CD8+ T ratio compared to the pre-treatment levels. Additionally, the level of CD8+ T cells was lower than the pre-treatment level. These findings suggest that hyperthermia can help balance the distribution of T cell subsets in peripheral blood. However, the reasons for the variations observed in different studies remain unclear. Further basic research is required in the future to elucidate the underlying mechanism of hyperthermia in regulating immune function. In our follow-up investigation, we discovered that the study group had a lower incidence of adverse reactions (such as myelosuppression, nausea, and vomiting) compared to the control group. Through literature review, we developed a hypothesis that heat therapy could stimulate the immune function of the body and provide feedback to the vomiting center in the brain. This feedback mechanism may trigger the release of endorphins, leading to the cessation of vomiting. Although we did not extensively study the impact of hyperthermia on hematotoxicity, we did observe a lower rate of myelosuppression in the hyperthermia group compared to the control group. Additionally, hyperthermia showed a tendency to improve chemotherapy-induced myelosuppression, which aligns with the findings of Wang Zhong et al.^22^. They also found that hyperthermia effectively promoted the mobilization of bone marrow granulocytes and counteracted the myelosuppressive effects of chemotherapy drugs.

Our study revealed a significant disparity in the composition and diversity of the intestinal microbiota in vivo between the two groups of lung cancer patients. Analysis of the 16S rRNA gene sequencing data indicated that the gut microbiota in the study group exhibited distinct alterations in biodiversity and composition compared to the control group. These findings suggest that hyperthermia has the potential to enhance the abundance and diversity of gut microbiota in lung cancer patients. Furthermore, animal model studies have demonstrated the direct impact of gut microbes on the response to immunotherapy^23,24,33,34^. In studies conducted on cancer patients undergoing immunotherapy, it has been observed that higher microbial diversity is associated with improved survival rates^25,26^. Previous research has indicated that microbial communities with a wide range of species are generally considered healthier, whereas decreased microbial diversity has been linked to various disease states^27^. Although our study did not specifically focus on immunotherapy, we conducted a literature review and discovered that gut flora can have a long-term impact on immune responses, which ultimately determines the effectiveness of immune checkpoint inhibitors (ICIs)^7,9,28,29^. Analyzing the progression-free survival (PFS) of the two patient groups statistically, we found a p-value of 0.059, which suggests a significant association, revealed that there were no statistically significant disparities in progression-free survival (PFS) between the groups exposed to hyperthermia treatment and those without it. These findings introduce intriguing possibilities concerning the potential mechanisms at play. One hypothesis is that hyperthermia could indirectly affect cancer treatment by altering the composition of the gut microbiota, potentially influencing the body's immune response or metabolic processes. Another possibility is that hyperthermia may have a direct impact on cancer cells, inducing cellular damage and apoptosis, which could enhance the efficacy of other treatment modalities. It is also conceivable that a combination of both factors, the influence on gut microbiota and direct cancer cell effects, could collectively contribute to the observed trends in PFS. Whereas the study did not establish statistical significance, it raises thought-provoking questions about the multifaceted ways in which hyperthermia may influence cancer treatment. Further research is essential to unravel the complex interplay among hyperthermia, gut flora, direct cancer cell effects, and their collective impact on patient outcomes. The mechanisms by which temperature affects gut microbes have been a subject of research. whereas the sensitivity of the gut barrier to temperature has been the primary focus, the exact ways in which heat stress alters gut permeability are not yet fully understood. Inflammation and hypoxia are important factors that can influence the levels of intestinal 'tight junction' (TJ) proteins such as occludin and occludin, as well as heat shock proteins and hypoxia-inducible factor (HIF)^30,31^. A study conducted by Se-Hoon Le et al.^32^examined the composition of fecal gut flora in 96 patients with non-small-cell lung cancer. They discovered that patients who responded well to treatment had a high abundance of Bifidobacterium bifidum in their fecal intestinal flora. In mouse models, researchers have discovered that certain strains can enhance the effectiveness of anti-Programmed Cell Death Protein 1 (ACP1) therapy by promoting the production of immune-stimulating molecules and metabolites, which in turn increase interferon-gamma production. Additionally, studies have shown that specific intestinal flora, such as Clostridiaceae and Ruminococcaceae, can accelerate the breakdown of estrogen in the intestine through the production of β-glucuronidase. This leads to higher levels of free estrogen, potentially contributing to the development of breast tumors^25,33^. On the other hand, the phylum Cyanobacteria and the phylum Reciprocal Bacteria, which are normally present in low numbers in the human body, exhibit a significant increase in lung cancer progression. According to the results of 16S rRNA gene sequencing, the abundance of g__Bifidobacterium was significantly increased in the study group after hyperthermia. However, the relative abundance of f__Ruminococcaceae and g__Faecalibacterium was high in the study group, and the mechanism behind this increase is currently unclear. In contrast, the abundance of p__Cyanobacteria and g__Faecalibacterium was significantly reduced in the control group after treatment, confirming the antitumor effect. The relative abundance of p__Cyanobacteria and g__Faecalibacterium was high in the control group, but the mechanism for this is not clear at present. This finding suggests that antitumor treatment can reduce the abundance of certain harmful bacteria and potentially control tumor progression.

This study's findings come with several important limitations that warrant careful consideration. Firstly, the sample size used in the study is relatively small, consisting of just twenty patients in each group. This limited sample size may not fully represent the broader population of individuals dealing with advanced, unresectable lung adenocarcinoma, potentially limiting the generalizability of the study's results to a larger and more diverse patient population. Additionally, the study's perspective is confined to a specific treatment protocol and patient population, which may limit its applicability to other cases of advanced lung adenocarcinoma or different cancer types. It's essential to acknowledge that cancer treatment outcomes are influenced by a multitude of variables, many of which were not thoroughly examined in this study.

The relationship between intestinal flora and tumors has been a widely studied area in recent years. Numerous preclinical and clinical studies have confirmed the connection between intestinal flora and antitumor immunity, as well as its impact on antitumor immunotherapy. However, the precise mechanism by which intestinal flora influences tumorigenesis, development, and antitumor immunotherapy remains unclear, necessitating further investigation at the molecular level. In order to enhance the effectiveness of antitumor immunotherapy, it is crucial to conduct extensive research on the beneficial bacteria in the intestinal flora. These studies aim to identify the specific advantageous flora for different clinical conditions and regulate the composition of the intestinal flora through various interventions such as antibiotics, fecal microbiota transplantation (FMT), probiotic interventions, and dietary modifications. This will help optimize the antitumor therapy and improve its outcomes. As basic and clinical research progresses, the emerging field of oncology hyperthermia will gain recognition among oncologists and establish its significance in integrative oncology. Consequently, it is crucial to conduct further research on the impact of hyperthermia for cancer and other diseases on the gut microbiota.

## Conclusion

Hyperthermia has a significant impact on improving the microecology of intestinal flora and the immunity of patients, but it does not have a significant effect on prolonging the progression-free survival of patients.

## Data Availability

Our data was tested and provided by Ovison Technologies, Ltd. and is non-public data, and Ovison Technologies did not provide us with detailed gene sequences, but only analysis results, which could not be uploaded to the database. The datasets used and/or analysed during the current study are available from the corresponding author on reasonable request.
